# Supplementary material for: Patterns of Intron Gain and Loss in Fungi
Source: PLoS Biol. 2004 Nov 30;2(12):e422. doi: 10.1371/journal.pbio.0020422 (PMC532390; doi:10.1371/journal.pbio.0020422)
Supplement: Table S1 — Also available at http://genes.mit.edu/NielsenEtAl/. (4.3 MB ZIP). [file pbio.0020422.st001.zip › NielsenEtAl/html/565.html]

AN6783.1.NCU04195.1.MG04868.1.FG07858.1


```
 CLUSTAL W (1.82) Multiple Sequence Alignments - Introns Inserted


Sequence 1: NCU04195.1	507 aa
Sequence 2: MG04868.1	555 aa
Sequence 3: FG07858.1	599 aa
Sequence 4: AN6783.1	496 aa
Alignment Length: 661 aa
Number Identitical Residues: 158 aa
Alignment Score (without introns) 9429


MG04868.1 	MPHDLNATSNTLRVLIPTS0PHQTRPAKLFRAQQAASMDVEKGRVPDNNVIAPASDQESV
NCU04195.1	-------------------~----------MVTQSDDNPLQKGHDVEANLQSIEADES--
FG07858.1 	-------------------~-----------------MTDTTNKTVPVRTMPPAVDNDD-
AN6783.1  	-------------------~-----------------MSAAEGSAAAESQSRPK------
          	                                          .                 

MG04868.1 	RPEMGSRAGRKSGSGNSTGRGSGGFLPTVLRFLNRAEARGIAPVPVEERTEKRAFNVFTL
NCU04195.1	----------KPAAPPSGLWGHICYYGHKVVVAGRIELQGISPIPVKERTVTKTLNIFTL
FG07858.1 	----------HDHDHSDVESLKAAALFHKALRMGRVEEKGIQPIPVEERTAARFYNIFTI
AN6783.1  	------------GSNSSFHFHQVPDKWLSRIANWGVELRGVTPVPIEERTDTRFINVFFV
          	                .                   * :*: *:*::***  :  *:* :

MG04868.1 	WW~TMNAN-ILA2ITFGMLAP-SFGLGLRDASLVIIFFSLLTAAPTSYLSTLGPKTGMRQ
NCU04195.1	WW~SMSCN-ILP2ITFGMLGP-VYGLSLRDSCLVILFFTLLSTLLPAYLCTLGPKLGMRQ
FG07858.1 	WA2EEIVDNENS~ITFGMLGPLVYGLSLRDSALIILFFCLFSTVGPAYLATFGPKTGMRQ
AN6783.1  	WF~TLSTN-LLP2IITGMVGTFVMGLSLRDASLIILFFNMLCTIPPAYFSTFGSRTGLRQ
          	*      :   . *  **:..   **.***:.*:*:** :: :  .:*:.*:*.: *:**

MG04868.1 	MVQARYSWG2RYLVTLPVILNLATLTGFCVVMAVIGGQCLSAVAGEGSLTPNVGIVIMAL
NCU04195.1	MIQARYSWG2RYIISLPVLLNLATMTGFSVIIVITGGQCLSAVAN-GHLSISVGIVIMSI
FG07858.1 	MIQARYSFG2RYLVSIPVLLNLATLTGFMVIIFVVGGQCLSAVSS-GHLSPDVGIVIIGI
AN6783.1  	MLHARFTFG~YYLVSIIVALNLCTIAGFGVVSSVLGGSTLAAVSG-GSIDDTAGIVVIGI
          	*::**:::*  *:::: * ***.*::** *:  : **. *:**:. * :   .***::.:

MG04868.1 	LSLFISFCGFRVLHFYERWASIPAVIAIVVATGCGGEGLKLQSTPP-VATVTAVFSFGMI
NCU04195.1	LTLIISFCGFTTLHMYERFAWIPAVIAIIVAVGCGGKYLHLQNPPADPPTPQGILSFAMI
FG07858.1 	LSLFISFCGFKVLHYYETYAFIPAIIAITIATGCGGSQLSKQATPTTPATASAVLSFGMI
AN6783.1  	CGMVVSFGGYKFLHQYERYCWLFALVAIVIATGVGGSKLSMQ-APTEPPSAATVLSFGGV
          	  :.:** *:  ** ** :. : *::** :*.* **. *  * .*.  .:   ::**. :

MG04868.1 	VASYMIPWA~ALASDFTTYLEPSTPA2IKIFAYSFAGLVTPPVLLMILGAAIAGAVPLNP
NCU04195.1	VASYMIPWA~CLASDFTTYLVPSTPS~WKIFTYSYTGLALPTILLMCLGAAIQGAIPSVP
FG07858.1 	VASYMIPWA~AIASDLTTYFDPKVPS~WRVFAYSYLGLVTPTILLMTLGAAIAGALPNVP
AN6783.1  	IAGFLIPWA0GMAADFSVYCTPSVSS~TRIFAYTYLGLFLPTVPLMALGAAIGSSTNSIS
          	:*.::****  :*:*::.*  *...:  ::*:*:: **  *.: ** ***** .:    .

MG04868.1 	TWEAGYDRNLVGGVLDAMLTPAGGFGKFLVVVLSFSLLGNLSATSYSVSLNLQQLFAPLT
NCU04195.1	AWSEAYNETLVGGVLQAMLAPAHGFGRFLVVILSFTLLGNLAATSYSITLNFQMLAP---
FG07858.1 	EWSAAYGETAVGGVLAAMLSSAGGFGKFVVVILSLTLLGNTGGTMYAITLNFQTLIPG--
AN6783.1  	SWSTGYTLYGVGGVLDAMLSPAGGFGKFVSVLLSFSLLGNLAASMYSISLNFQLLLPWK-
          	 *. .*    ***** ***:.* ***:*: *:**::**** ..: *:::**:* * .   

MG04868.1 	RGSEILKRVPRYAYTILLTALVIPIGIRAAQDFFVSLENFLGLIGYWSAAFVSLLMVEHL
NCU04195.1	----VLFKVPRYLFAVILAAILIPVSIKAAADFFDSLENFVSLIGYWSSAFLGVVMVDHL
FG07858.1 	-----LIKIPRYAFAVVVTVIVIPTALRAQRDFFVNLENFVALIGYWSASFIGIVTVEHL
AN6783.1  	----LWRRVPRFVWSIIYTGVLIPVSVVAAKSFFTNLENFLYVIAYWSAGFFSVVTVEHF
          	       ::**: :::: : ::** .: *  .** .****: :*.***:.*..:: *:*:

MG04868.1 	WFRAGDCDSYDP-------AIWNDGARLPIGVAALVSSVLSFLLVVPSMSQVWWTGPIAR
NCU04195.1	WEKKGDCGRYDL-------EAWNDASKLPWGVAALTASVASFALVIPSMDQVWWQGPIAK
FG07858.1 	VFRKGRYESYDH-------AIWNVASELPLGIAAIAAGIICYALVVPCMAQAWWTGPIAK
AN6783.1  	VFRQGAFDTYTADPSGDGKTTWDNQSKLPTGLAAIGAMALSFGLVIPCMGQVWFTGPLAK
          	  : *    *  ..:....  *:  :.** *:**: :   .: **:*.* *.*: **:*:

MG04868.1 	VTGDLG~-----FEFAFVVTGL~L~YVPFRTLEKHLTKR-----~---------------
NCU04195.1	TTGDLG~-----FEFAFVVSGL~L~YPPLRMVEKKISGR-----~---------------
FG07858.1 	TTGDIG2SCTRHFESTTISTRI0A~SLLAKAAEKQYPQTPRQRL2HYHNYLEHAHSDVLP
AN6783.1  	TTGDIG~-----FEIALVLAGA~L2P-YLRGVD-----------~---------------
          	.***:*      ** : : :         :  :                           

MG04868.1 	------------------------------------------------------------
NCU04195.1	------------------------------------------------------------
FG07858.1 	RTPPPVSPKGAKVPRPWMRFVLTEPSFGSKVYGLRVYVWIVVYAVSVGHYDEIVVDGEMA
AN6783.1  	------------------------------------------------------------
          	                                                            

MG04868.1 	-----------
NCU04195.1	-----------
FG07858.1 	ALVVNSCARRT
AN6783.1  	-----------
          	
```
